# Deciphering salivary microbiome signature in Crohn’s disease patients with different factors contributing to dysbiosis

**DOI:** 10.1038/s41598-023-46714-8

**Published:** 2023-11-06

**Authors:** Hala Elzayat, Talha Malik, Haifa Al-Awadhi, Mazen Taha, Gehad Elghazali, Farah Al-Marzooq

**Affiliations:** 1 Department of Medical Microbiology and Immunology, College of Medicine and Health Sciences, United Arab Emirates University, P.O. Box 15551, Al Ain, UAE; 2 Department of Medicine, Sheikh Shakhbout Medical City, Abu Dhabi, UAE; 3 Department of Pediatric Gastroenterology, Tawam Hospital, Al Ain, UAE; 4 Department of Internal Medicine, Tawam Hospital, Al Ain, UAE; 5 Department of Immunology, Sheikh Khalifa Medical City, Union71-Purehealth, Abu Dhabi, UAE; 6 Zayed Center for Health Sciences, United Arab Emirates University, Al Ain, UAE

**Keywords:** Microbiology, Biomarkers, Gastroenterology

## Abstract

Crohn's disease (CD) is a chronic inflammatory bowel disease. An imbalanced microbiome (dysbiosis) can predispose to many diseases including CD. The role of oral dysbiosis in CD is poorly understood. We aimed to explore microbiome signature and dysbiosis of the salivary microbiome in CD patients, and correlate microbiota changes to the level of inflammation. Saliva samples were collected from healthy controls (HC) and CD patients (n = 40 per group). Salivary microbiome was analyzed by sequencing the entire 16S rRNA gene. Inflammatory biomarkers (C-reactive protein and calprotectin) were measured and correlated with microbiome diversity. Five dominant species were significantly enriched in CD, namely *Veillonella dispar*, *Megasphaera stantonii*, *Prevotella jejuni*, *Dolosigranulum pigrum* and *Lactobacillus backii*. Oral health had a significant impact on the microbiome since various significant features were cariogenic as *Streptococcus mutans* or periopathogenic such as *Fusobacterium periodonticum.* Furthermore, disease activity, duration and frequency of relapses impacted the oral microbiota. Treatment with monoclonal antibodies led to the emergence of a unique species called *Simonsiella muelleri.* Combining immunomodulatory agents with monoclonal antibodies significantly increased multiple pathogenic species such as *Salmonella enterica, Escherichia coli, Klebsiella pneumoniae* and *Pseudomonas aeruginosa.* Loss of diversity in CD was shown by multiple diversity indices. There was a significant negative correlation between gut inflammatory biomarkers (particularly calprotectin) and α-diversity, suggesting more inflammation associated with diversity loss in CD. Salivary dysbiosis was evident in CD patients, with unique microbiota signatures and perturbed species that can serve as disease biomarkers or potential targets for microbiota modulation. The interplay of various factors collectively contributed to dysbiosis, although each factor probably had a unique effect on the microbiome. The emergence of pathogenic bacteria in the oral cavity of CD patients is alarming since they can disturb gut homeostasis and induce inflammation by swallowing, or hematogenous spread of microbiota, their metabolites, or generated inflammatory mediators.

## Introduction

Inflammatory bowel diseases (IBD) are chronic idiopathic relapsing inflammatory disorders of the gastrointestinal tract (GIT)^1^. Crohn’s disease (CD) is a chronic IBD involving mostly the terminal ileum and the large intestine; however, transmural inflammation may involve any segment of the GIT from mouth to anus^1^. The precise etiology and pathogenesis of CD remain unclear, but the most accepted hypothesis suggests a complex interaction between genetics, environmental factors, and the immune system leading to an aberrant immune response and chronic intestinal inflammation^2^. The initiating stimuli for immune dysregulation in CD are not fully explained by genetic predisposition. Genetic loci associated with CD development can alter the delicate relationship between microbiota and host leading to a dysregulated immune response and intestinal inflammation^3^. In a meta-analysis of IBD genome-wide association in more than 75,000 cases and controls, many genes associated with CD were found responsible for immune system recognition of microbes, suggesting that IBD patients are unable to either nurture beneficial bacteria or destroy the harmful ones^4^.

Most microbiome studies used next-generation sequencing (NGS) techniques targeting a specific variable region of the 16S rRNA gene by short-read sequencing; thus, they stopped at the phylum or genus levels^5^. On the contrary, third-generation sequencing produces long reads; thus, it is more reliable for exploring the microbiome in depth down to the species level^6^.

Several studies reported the importance of gut microbiome and its relation to CD, with the potential for use as biomarkers for disease status and response to therapy^7–9^. The oral microbiome is second to the gut microbiome in size. Genomic sequencing has identified intestinal enrichment of oral-associated bacteria and concluded that the gut microbiome can cross-talk with the salivary microbiome, possibly having a role in CD pathogenesis^10,11^. Pathological changes to the oral microbiota, such as those occurring during periodontal disease, are associated with multiple inflammatory conditions, including IBD^12^. Whether this association is a consequence of oral inflammation or because oral bacteria can directly drive inflammation at distal sites remains under debate^10^. The reasonable concentration of IBD research on the intestinal microbiota has left a lot to be discovered about the oral microbiome. Limited studies in the past decade addressed the relationship of salivary microbiome to CD and its link to gut dysbiosis^2,13,14^. It is important to consider that the oral microbiome can be affected by many factors possibly contributing to dysbiosis, such as patients’ oral health and medications consumed. However, there is no previous study exploring various factors correlating with the microbiome alterations in CD patients. Elucidating different factors might lead to a better understanding of CD pathogenesis and help in identifying key microbiota that can be used as diagnostic or prognostic biomarkers, or even as targets for therapeutic microbiota modulation. This study was conducted to explore the alterations in the salivary microbiome in CD patients at different taxonomic levels, with a focus on species. Our aim was to characterize the compositional changes in the salivary microbiota of patients with CD compared to healthy controls (HC). We also aim to compare CD patients for salivary microbiome complexity and diversity according to different variables, including oral health, IBD drug use, disease duration, activity of the disease and frequency of relapse of symptoms.

## Methods

### Ethical approval

Ethical approval was obtained from Abu Dhabi Health Research and Technology Ethics Committee, Department of Health, Abu Dhabi, United Arab Emirates (UAE); approval number: DOH/CVDC/2020/2470. Subjects were recruited from Sheikh Shakhbout Medical City, Abu Dhabi and Tawam Hospital, Al Ain, UAE from August 2021 to February 2022. All participants signed an informed consent prior to enrollment in the study. The study was conducted in compliance with national and international standards including Helsinki declaration. All methods and procedures were performed in accordance with the relevant guidelines and regulations.

### Study design and patients

This is a case–control study including 80 subjects, 40 CD patients and 40 age-gender matched healthy controls (HC). Patients with established CD (based on endoscopic, histopathologic, radiologic and clinical presentation), were recruited. Crohn’s Disease Activity Index (CDAI) was calculated^15^. Other information related to CD were recorded including age at onset of the disease, duration, frequency of relapses, and medications received.

HC were subjects with no known chronic inflammatory disorders, no GIT complaints or were otherwise healthy. Subjects using antibiotics one month prior to enrollment were excluded as this can affect the oral microbiome^16^. Pregnant and lactating ladies, as well as all participants having serious systemic diseases and smokers were also excluded from the study. Oral examination was done by the same dentist to check for dental caries and periodontal diseases for all CD and HC subjects.

## 3Saliva sample collection, processing, and DNA extraction

One hour before sample collection, participants refrained from drinking, eating, and cleaning their teeth. To eliminate any food residue, the mouth was rinsed with water prior to sample collection. At least 2 ml of unstimulated whole saliva was collected into a sterile container^17^, then 1 ml of each sample was transferred into a sterile tube, and centrifuged to pellet the cells. The pellet was used for DNA extraction while the supernatant was removed carefully and stored at − 80 °C for later salivary biomarkers analysis^17^. DNA was extracted using Wizard® HMW DNA extraction kit (Promega, USA). A nanodrop (Thermo Scientific, USA) was used to verify DNA quality and quantity.

### Salivary microbiome profiling

The full-length bacterial 16S rRNA gene (1500 bp) was sequenced with Mk1C Oxford Nanopore sequencer using 16S Barcoding kit 1-24 (SQK-16S024; Oxford Nanopore Technologies, UK)^18^. DNA was amplified by PCR using LongAmp™ Taq 2 × Master Mix (New England Biolabs, UK), and purified by AMPure XP beads (Beckman Coulter, USA), then quantified by Qubit 2 (Thermo Scientific, USA). Equimolar amounts of PCR products were pooled, and sequenced using R9.4 flow cells (Oxford Nanopore Technologies, UK), as described before^19,20^. MinKNOW software (Oxford Nanopore Technologies, UK) was used for live base-calling and data acquisition. Raw data were converted into FASTQ using Guppy, followed by demultiplexing, and removal of nanopore adaptor sequences with a default minimum Q score of 9. Reads were analyzed by Kraken taxonomic sequence classification system using Partek® Genomics Suite® software (Copyright^©^ 2022; Partek Inc, USA). Operational Taxonomic Units (OTUs) were analyzed using Microbiome Analyst 2.0 platform (McGill, Canada)^21^, which was also used for other downstream analyses. Linear discriminant analysis (LDA) effect size (LEfSe) was used to detect biomarkers from microbial profiles, at an LDA threshold of 2^18,22^. For α-diversity, different indices were used including Shannon and Simpson diversity (richness, and evenness measures), Chao1 (estimator for diversity from abundance data), ACE (Abundance-based Coverage Estimator), and Observed Species (counts of unique OTUs per sample). For β-diversity (inter-group comparison), Bray–Curtis and Jaccard distance matrices were used to assess the dissimilarity of samples, visualized through principal coordinate analysis (PcoA), and compared using permutational multivariate ANOVA (PERMANOVA)^21^. A Venn diagram was generated to identify the shared OTUs among CD patients grouped based on multiple factors using InteractiVenn tool^23^.

### Enzyme-linked immune-sorbent assay (ELISA) for inflammatory biomarkers

Quantification of salivary C-reactive protein (CRP) and calprotectin (CAL) was done using ELISA kits (MBS2505217 and MBS7606803, MyBioSource, USA, respectively) according to the manufacturer’s protocol. For CD patients, serum CRP and fecal CAL were obtained from the patients’ electronic records as these tests are done routinely for IBD patients.

### Statistical analyses

SPSS Statistics (version 28; IBM SPSS® Statistics, USA) was used for data analysis. Mann–Whitney U or Kruskal–Wallis tests were used for the comparison of microbiota relative abundances, inflammatory biomarkers, and α-diversity indices. Statistical significance was determined at *p* < 0.05. Graphs were generated using Microbiome Analyst 2.0 platform (McGill, Canada), GraphPad Prism® Version 9.4.0 (GraphPad Software, USA) and R (version 4.1.2) which was used for heatmap construction by pheatmap package. For correlation analysis, corrplot package was used for the calculation and graphical presentation of correlation coefficients, for numerical variables including inflammatory biomarkers, α-diversity indices, CDAI and disease duration.

## Results

### Study population

A total of 40 CD patients and 40 HC were recruited. Males with CD were more than females (60% and 40%, respectively). The age of CD patients was matching to controls ± 2 years difference. The age range was between 16–52 years for CD and 18–54 years for HC with mean ± SD of 32.75 ± 10 and 33.37 ± 9.67 years for CD and HC, respectively. CD participants’ characteristics are shown in Table 1.

**Table 1:** Characteristics of CD participants (n = 40).

Characteristics	% (n)
Duration of the disease	
Newly diagnosed	15 (6)
1–10 years	52.5 (21)
> 10 years	32.5 (13)
Number of relapses	
0–1 times per year	72.5 (29)
≥ 2 times per year	27.5 (11)
Activity of the disease	
Active-relapse (CDAI * ≥ 150)	25 (10)
Inactive-remission (CDAI < 150)	75 (30)
Oral health ^#^	
Dental caries	12.5 (5)
Periodontal disease	20 (8)
Caries with periodontal disease	40 (16)
Good oral hygiene	27.5 (11)
IBD drugs	
Monoclonal antibodies alone	62.5 (25)
Monoclonal antibodies + steroids	22.5 (9)
Monoclonal antibodies + immunosuppressants	7.5 (3)
Monoclonal antibodies + steroids + immunosuppressants	7.5 (3)

*CDAI: Crohn’s disease activity index.

^#^All the factors above are unique for CD as they are related to disease manifestations or therapy used for CD. Oral health was a shared factor for CD and HC. For oral health in HC, it includes good oral hygiene (n = 21; 52.5%), dental caries (n = 2; 5%), periodontal disease (n = 4; 10%), and caries with periodontal disease (n = 13; 32.5%).

As shown in Table 1, most CD patients were diagnosed in the past 1–10 years (52.5%), while less patients were newly diagnosed (15%). The majority of patients (75%) had inactive disease (CDAI < 150), while one quarter (25%) had active disease (CDAI ≥ 150). As most patients had inactive disease, 72.5% experienced less frequent relapses of symptoms including none or one relapse per year, and less patients (27.5%) had ≥ 2 relapses per year. Relapse of CD symptoms included abdominal pain, blood in stool, diarrhea, and weight loss. Since most patients were interviewed in the infusion clinic, the majority were receiving intravenous medications, particularly monoclonal antibodies (mainly infliximab; 62.5%), but few used other monoclonal antibodies such as vedolizumab, ustekinumab and adalimumab. Other medications including steroids (prednisolone, hydrocortisone, and budesonide) and immunosuppressants (azathioprine and mercaptopurine) were taken concurrently to reduce symptoms in some patients.

As for oral health, most CD patients had poor oral health (72.5%) compared to HC (47.5%). In CD, poor oral health is further classified into caries (C) (12.5%), periodontal disease and caries (P + C) (40%), or periodontal disease alone (P) (20%), while some CD patients (27.5%) had good oral health (H). Noteworthy, none of the patients had oral manifestations of CD (i.e., cobblestoning or oral ulcers). As for HC, most of them had good oral health (52.5%), while others had P + C (32.5%), P (10%) or C (5%).

### Bacterial 16S rRNA sequencing

The average read length was 1,542.68 ± 24.9 bp which is equivalent to the length of the bacterial 16S rRNA gene. Comparison of taxa detected in 40 CD and 40 HC was done. At the phylum level, *Firmicutes/Bacteroidetes* ratio was reduced in CD (12.5 ± 10.46) compared to HC (19.7 ± 22.36), but the difference was not statistically significant (*p* > 0.05). LEfSe analysis indicated significant differences between CD and HC. *Tenericutes* and *Spirochetes* phyla were significantly more in HC and depleted in CD. Genus *Dolosigranulum* was significantly higher in CD and depleted in HC, while 16 genera were significantly higher in HC and depleted in CD.

**Figure 1:**
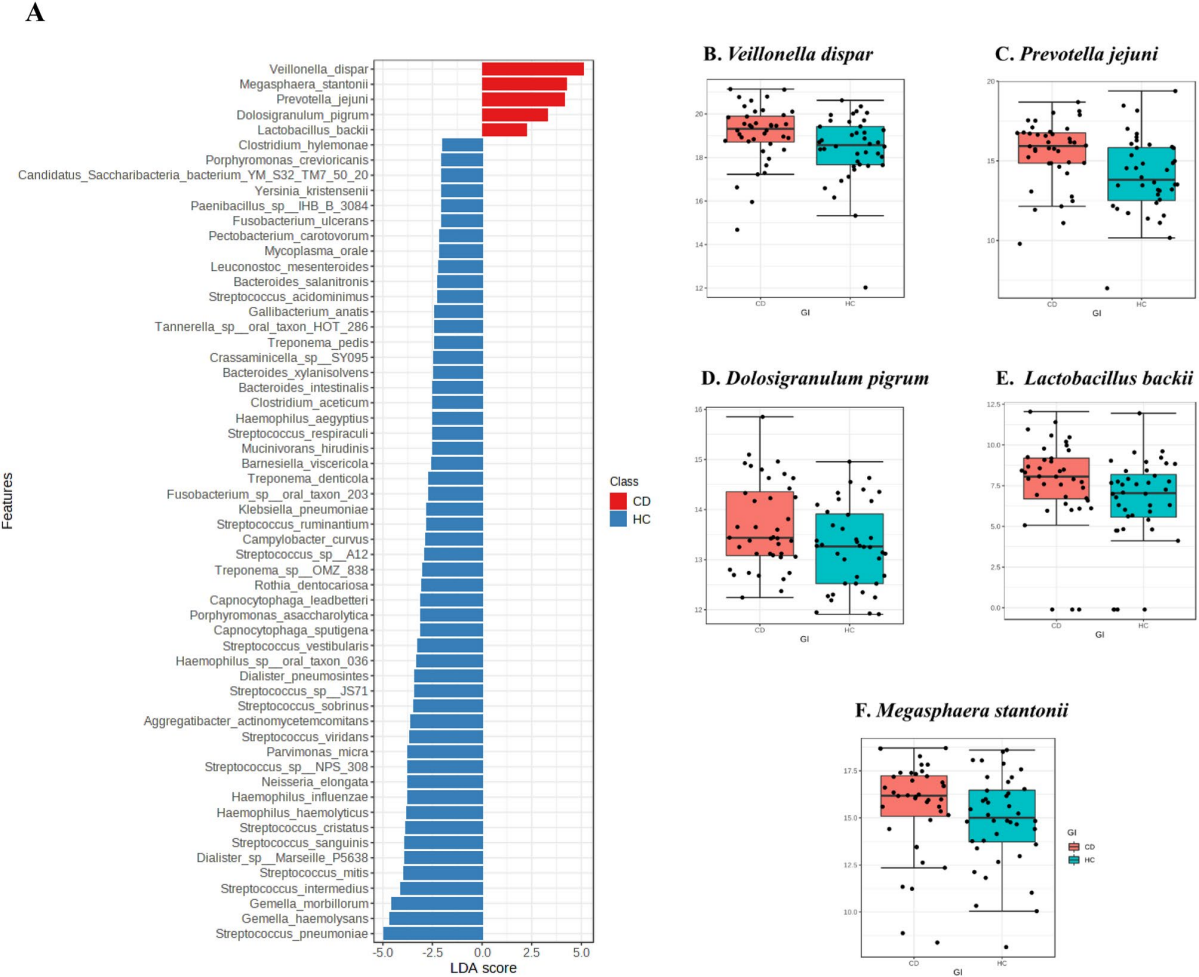
Significant features at the species level detected using LDA in CD compared to HC (**A**). Blue bars represent taxa in higher proportions in the healthy group; red bars represent taxa in higher proportions in CD participants, with an LDA score ≥ 2. Significant bacterial species detected in the saliva of CD patients in higher abundance compared to HC are shown in (**B**–**F**). Log transformed microbial counts are shown.

At the species level, LDA analysis clearly demonstrated 65 significant features (Fig. 1A), five of which were significantly higher in CD, and the remaining 60 were significantly more in HC and depleted in CD. These five dominant species are also shown in Fig. 1B–F, demonstrating the significant difference in the relative abundance of these key microbiota in CD compared to HC.

### Factors contributing to dysbiosis in CD

Patients with CD (n = 40) were compared for microbiota composition based on different factors that might contribute to dysbiosis, as follows:

### Oral health

**Figure 2:**
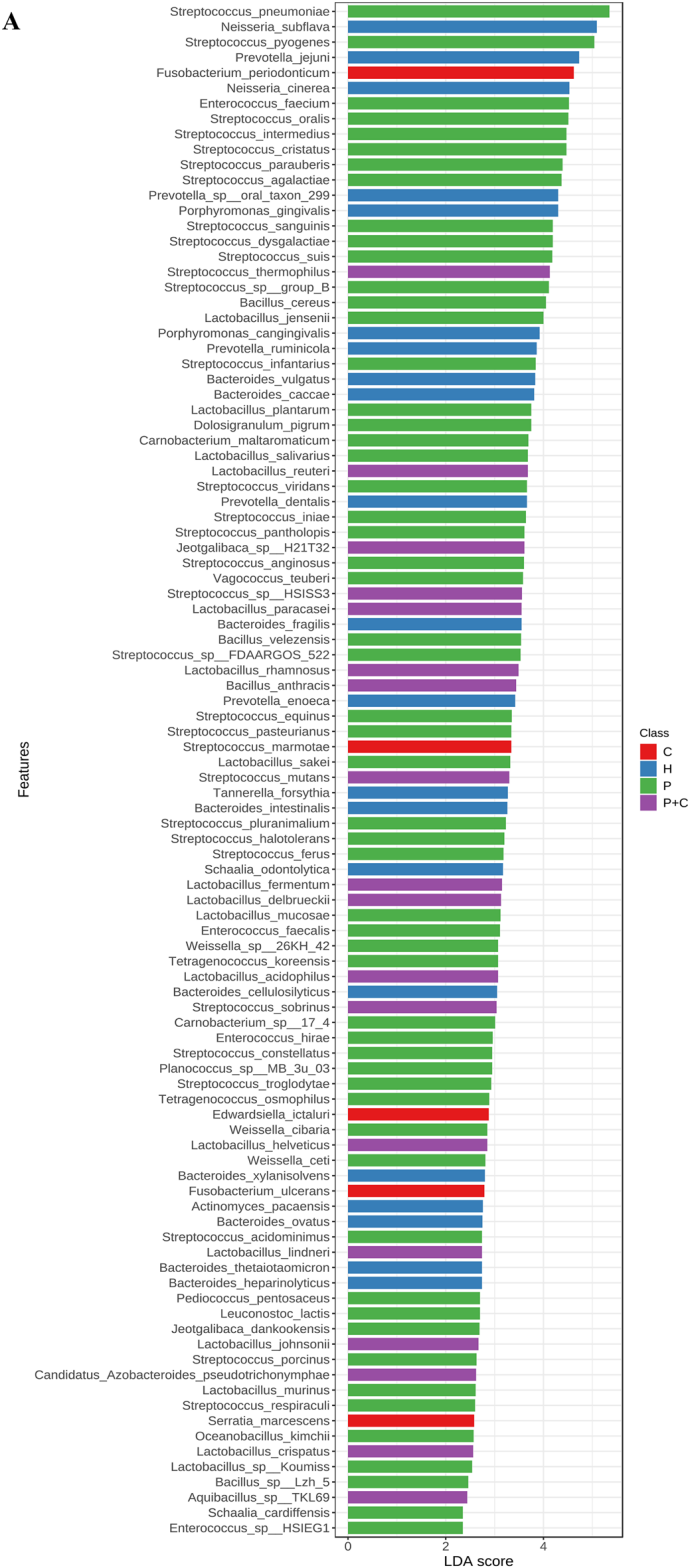

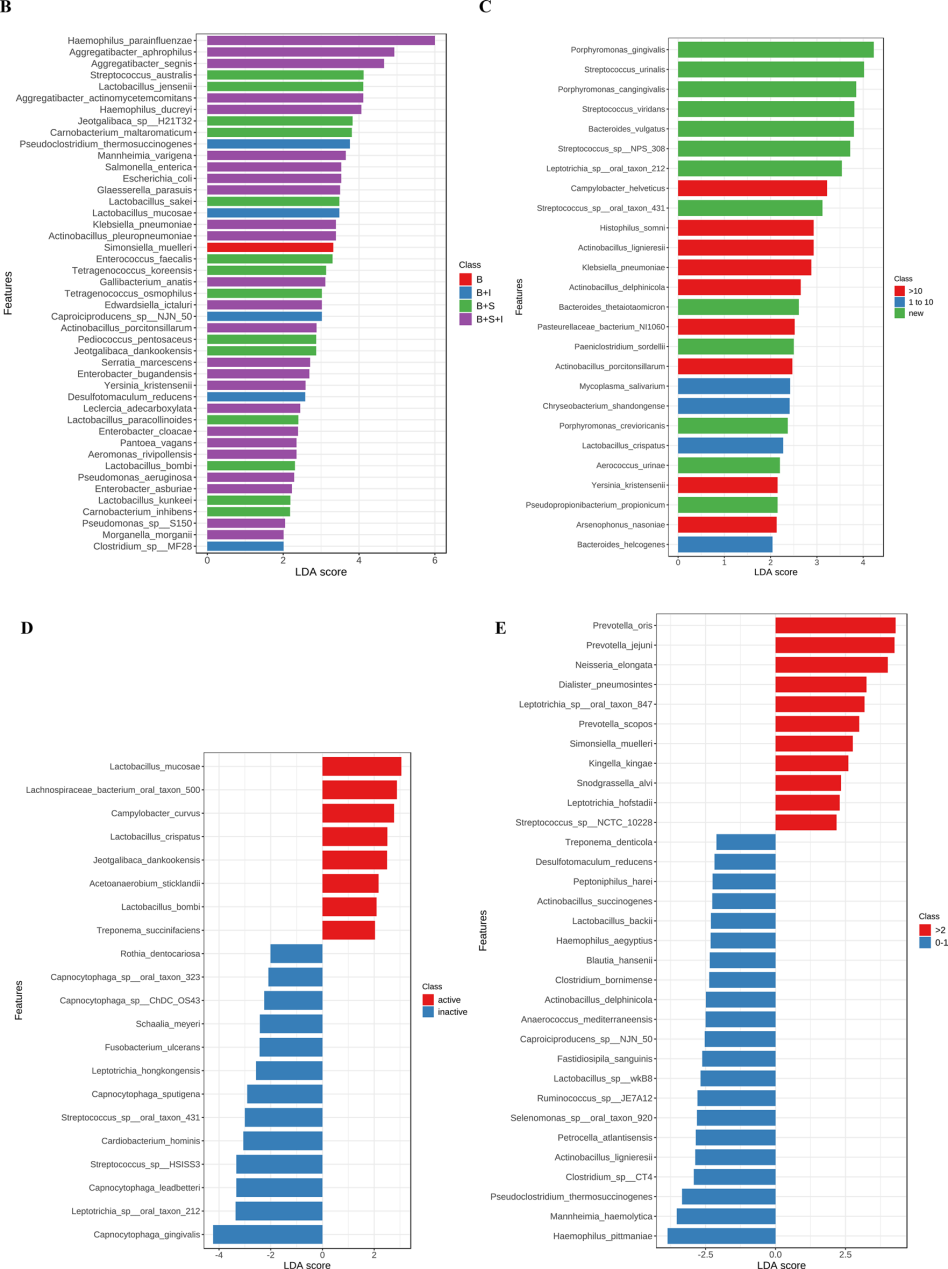
Significant features at the species level detected using LDA in CD patients based on variation in oral health status (**A**), IBD drugs (**B**), disease duration (**C**), activity (**D**), and frequency of relapses (**E**). Species with LDA score ≥ 2 are shown.

As shown in Fig. S1-A, two significant phyla were detected (*Fusobacteria* in H and *Actinobacteria* in P), in addition to 28 significant genera, mainly *Bacteroides* (H), *Fusobacteria* (C), *Streptococcus* (P) and *Lactobacillus* (P + C) with the highest LDA scores (*p* < 0.05). At the species level (Fig. 2A), 121 significant features were recognized with LDA score ≥ 2, including *Neisseria subflava, Tanerella forsythia, Porphyromonus gingivalis, Prevotella jejuni, Prevotella dentalis, Prevotella enoeca, Bacteroides fragilis,* and *Bacteroides intestinalis* in H*, Fusobacterium* such as *F. periodonticum* and *F. ulcerans* in C, *Streptococci* such as *S. mutans, S. pyogenes, S. oralis*, and *S. viridans* in P, with multiple significant species such as *Streptococcus mutans*, *Lactobacillus fermentum* and *Lactobacillus acidophilus* in patients with P + C.

### IBD medications

At the phylum level, *Proteobacteria* were higher in patients receiving three medications (monoclonal antibodies, steroids and immunosuppressants). A total of 21 significant bacterial features were detected at the genus level in CD patients receiving IBD drugs as shown in Fig. S1B. Interestingly, a sole and significant bacteria appeared in patients receiving only monoclonal antibodies as the main treatment for CD which was the genus *Simonsiella.* The other 20 bacterial profiles were miscellaneous, but mainly detected in patients receiving the combination of three types of medications with notable genera such as *Haemophilus, Aggregatibacter, Actinobacillus, Salmonella, Serratia, Enterobacter, Escherichia,* and *Pseudomonas.* At the species level, a total of 45 significant features were detected in patients receiving IBD medications (Fig. 2B). The distinctive bacteria of great interest (*Simonsiella muelleri*) appeared in patients receiving only monoclonal antibodies without any other medication. Most significantly increased bacteria were detected in patients receiving three types of medications, including *Escherichia coli, Salmonella enterica, Klebsiella pneumoniae*, *Pseudomonas aeruginosa, Enterobacter cloacae, Haemophilus parainfluenzae,* and *Aggregatibacter actinomycetemcomitans,* among others.

### Duration of disease

At the phylum level, no significant bacterial features were detected. At the genus level, a total of eight significant features were detected. *Porphyromonas* was the dominant genus in newly diagnosed patients, while *Pasteurella* was dominant in patients who had the disease for more than a decade, as shown in Fig. S1–C. At the species level (Fig. 2C), 13 bacteria were in patients newly diagnosed with CD, including multiple species from the genus *Porphyromonas* such as *P. gingivalis* and *P. cangingivalis,* and from the genus *Streptococcus* such as *S. urninalis* and* S. viridans.* Only four species were detected in patients having the disease for 1–10 years; while nine species were detected in patients having CD for more than 10 years, including some pathogenic bacteria such as *Klebsiella pneumoniae.*

### IBD activity

At the phylum level, no significant bacterial features were detected. At the genus level, six dominant features were detected, two of which are *Acetoanaerobium* and *Mycoplasma* in patients with active disease and four genera (*Schaalia, Cardiobacterium, Leptotrichia,* and *Capnocytophaga*) were significantly higher in patients with inactive CD, as shown in Fig. S1-D. At the species level (Fig. 2D), a total of 22 significant features were detected, eight of which were in patients with active disease, including species from the genus *Lactobacillus* (*L. mucosae, L. crispatus* and *L. bombi*). Another 13 species were detected in patients with inactive CD, five of which belong to the genus *Capnocytophaga*, in addition to other species such as *Fusobacterium ulcerans.*

### Frequency of relapse of symptoms

At the phylum level, no significant bacterial features were detected. At the genus level, 15 significant features were detected, most common are *Eikenella, Simonsiella* and *Kingella* in patients who had relapses more than twice a year, as shown in Fig. S1-D. Multiple species were found in patients who had relapses up to once per year, but importantly, there was an increase in genus *Clostridium,* among others. At the species level (Fig. 2E), 11 bacterial species were found in patients having a relapse more than twice a year. The most common species are *Prevotella oris, Prevotella jejuni* and *Simonsiella muelleri.* In patients with less frequent relapse of symptoms (0–1 per year), 21 significant bacterial species were found, including multiple species of *Clostridium, Lactobacillus* with *Ruminococcus.*

### Interaction between different factors contributing to dysbiosis in CD 

**Figure 3:**
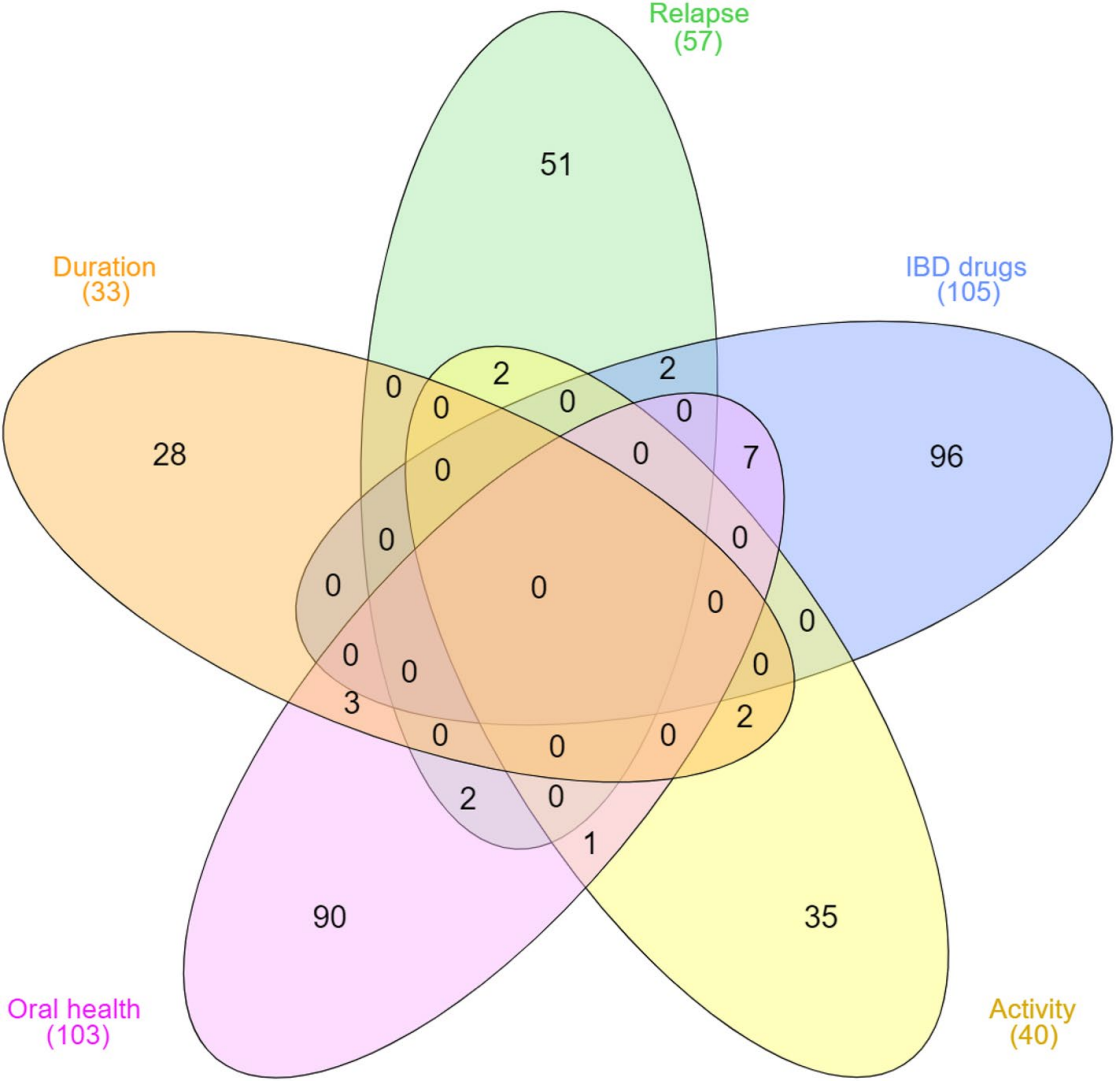
Venn diagram of exclusive and shared taxonomically unique microbiota at the species level based on five factors: oral health, IBD drugs, disease duration, relapse, and activity. Overlaps also are shown that demonstrate the shared microbial species within the groups.

A Venn diagram (Fig. 3) was generated to identify shared species in CD patients grouped according to different factors that may affect the microbiome, including oral health, IBD drug use, activity of the disease, relapse of symptoms and disease duration. Significantly altered species (identified by LDA analysis) were unique to each factor. No species were shared among the five factors. Few species (n = 0–7) were common between some factors. When factors related to the disease were considered, two species were common between relapse and IBD drugs (*Simonsiella muelleri* and *Hungatella hathewayi*); two species were common between duration and activity (*Leptotrichia sp.* oral taxon 212 and *Kluyvera intermedia*); and two species were common between relapse and activity (*Lactobacillus zymae* and *Treponema primitia*). As for oral health relation to the other CD factors, seven common species were shared with IBD drugs (*Thalassolituus oleivorans, Aquisphaera giovannonii, Acinetobacter pittii, Photobacterium damselae, Lactobacillus ginsenosidimutans, Lactobacillus jensenii*, and *Enterococcus durans*); three shared with duration (*Streptomyces sp Mg1, Porphyromonas cangingivalis* and *Bacillus asahii*); two shared with relapse (*Pseudomonas synxantha* and *Actinomyces oris*) and one shared with activity (*Lactobacillus nagelii*).

**Figure 4:**
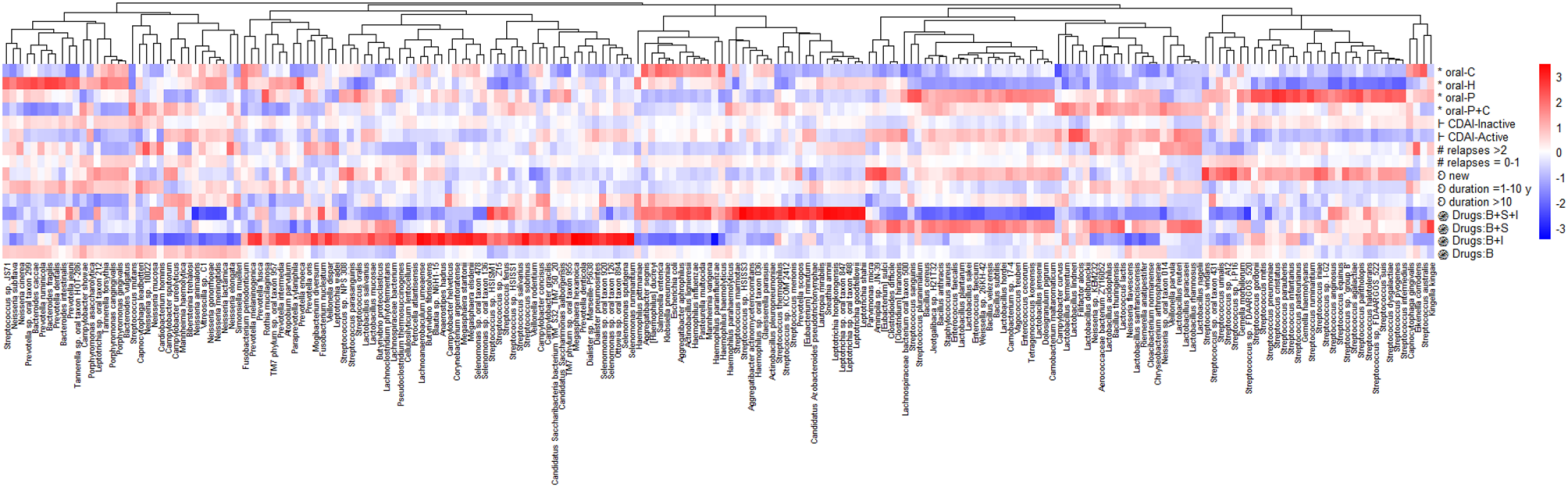
Distribution of different species (relative abundance ≥ 0.1%) in CD patients grouped according to oral health status (marked with *), activity (marked with ⊢), frequency of relapses (marked with #), disease duration (marked with ), and IBD drugs (marked with ). Red color indicates high abundance in a particular group and blue color indicates low abundance. Dendrograms show the clustering of different species in all the groups.

When a heatmap was constructed for the species with abundance ≥ 0.1% (Fig. 4), microbiota signature was obvious in each group of patients differentiated based on the five factors. As shown in Fig. 4, microbiota species depleted or enriched in each subgroup were very unique for each factor, and different from the species altered by the other factors. The most striking variations were noted among patients grouped according to oral health status and medications (marked by * and , respectively in Fig. 4).

### Microbiome diversity 

**Figure 5:**
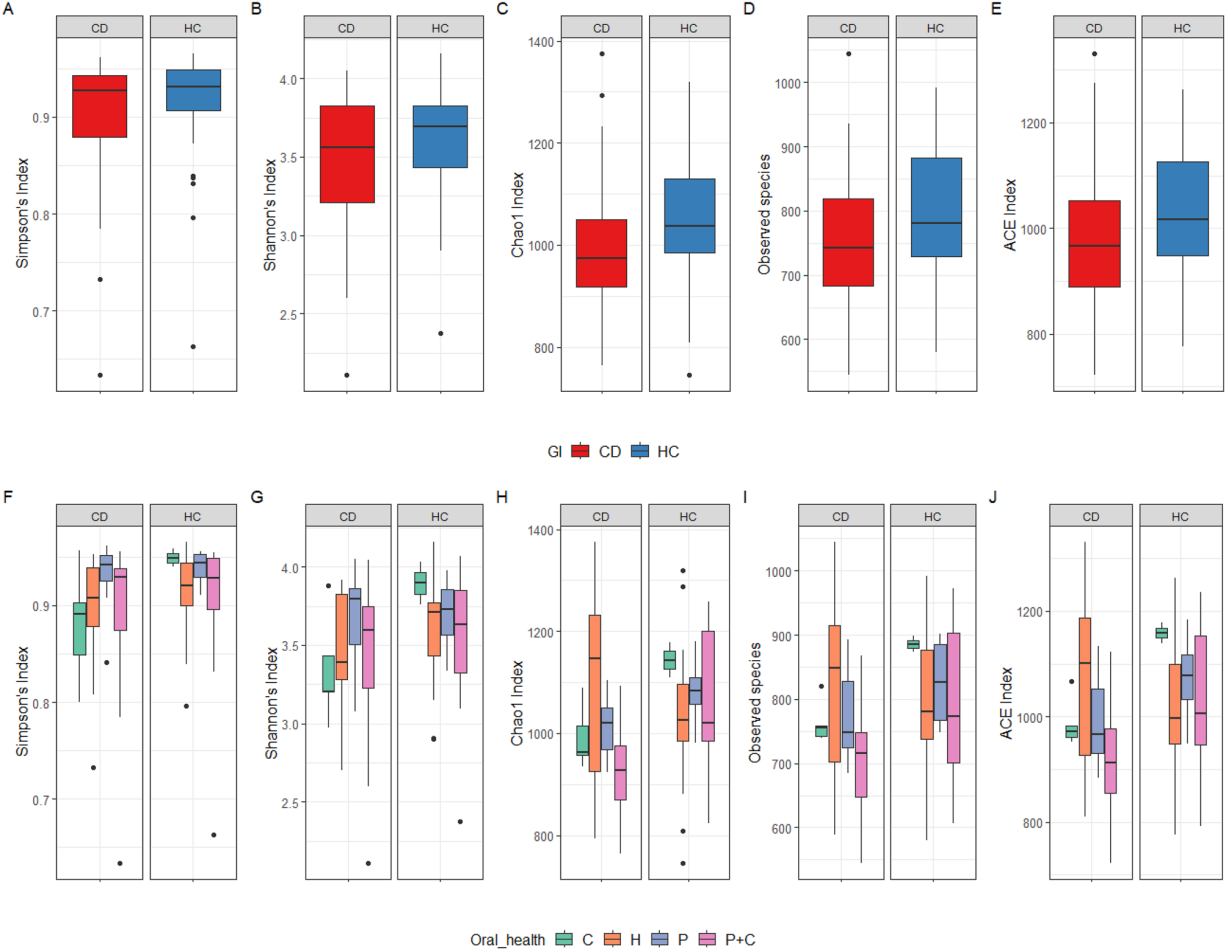
Alpha diversity indices for comparison of CD and HC salivary microbiome. (**A**–**E**) show alpha diversity indices, for comparison of the salivary microbiome in the participants grouped based on the gastrointestinal (GI) health status (CD and HC), while (**F**–**J**) show alpha diversity indices based on the oral health status in each group. For oral health: healthy (**H**), caries (C), periodontitis (P) and periodontitis and caries (P + C).

Α-diversity indices were compared between CD and HC, revealing significant differences in observed species, Chao 1 and ACE (*p* values: 0.049, 0.022 and 0.048, respectively), while Simpson’s and Shannon indices were not significantly different (*p* values > 0.05). The results showed a significant reduction in α-diversity in CD compared to HC (Fig. 5A–E).

Comparison of α-diversity indices in CD patients based on oral health (Fig. 5F–J) indicated that patients with poor oral health had less diverse microbiome and lower indices with significant differences in observed species, Chao 1 and ACE (*p* value: 0.048, 0.025 and 0.025, respectively). Patients with P + C had the lowest values, while Simpson’s and Shannon indices were not significantly different (*p* values > 0.05). As for HC, a comparison of α-diversity indices showed non-significant differences among the groups (Fig. 5F–J). Non-significant difference was found in α-diversity when CD patients were compared based on other factors including IBD medications, disease activity, duration and frequency of relapse of symptoms, as shown in the supplementary data (Figs. S2–S5).

**Figure 6:**
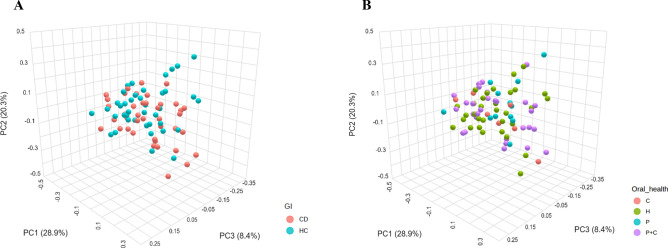
Beta diversity represented by principal coordinates analysis in CD patients compared to HC (**A**), and in all the participants grouped based on oral health status (**B**).

As shown in Fig. 6A, β-diversity demonstrated non-significant dissimilarity across CD and HC (*p*-value: 0.067) using Bray Curtis and Jaccard as the distance methods with principal coordinates analysis (PCoA). Non-significant difference in β-diversity was noted when all the 80 subjects were compared based on oral health (Fig. 6B), as no distinct cluster was identified.

A non-significant difference in β-diversity was found in CD patients compared based on different factors including oral health, IBD medications, disease activity, duration, and frequency of relapse of symptoms, as shown in the supplementary data (Fig. S6).

### Inflammatory biomarkers 

Among the 80 saliva samples, CAL values were more than CRP (3.48 ± 3.22 and 1.12 ± 1.714 ng/ml, respectively), and their levels were non-significantly correlated. In CD patients, salivary CRP and CAL were more than those in HC, but the difference was non-significant. Salivary CRP showed non-significant difference (*p* > 0.05) among patients with different oral health conditions. For salivary CAL, subjects with caries had significantly higher levels (6.39 ± 4.48 ng/ml) compared to patients with periodontal disease (2.34 ± 2.9 ng/ml) with a *p*-value: 0.009. The difference in all inflammatory markers was non-significant for the other groups and other factors in CD patients.

**Figure 7:**
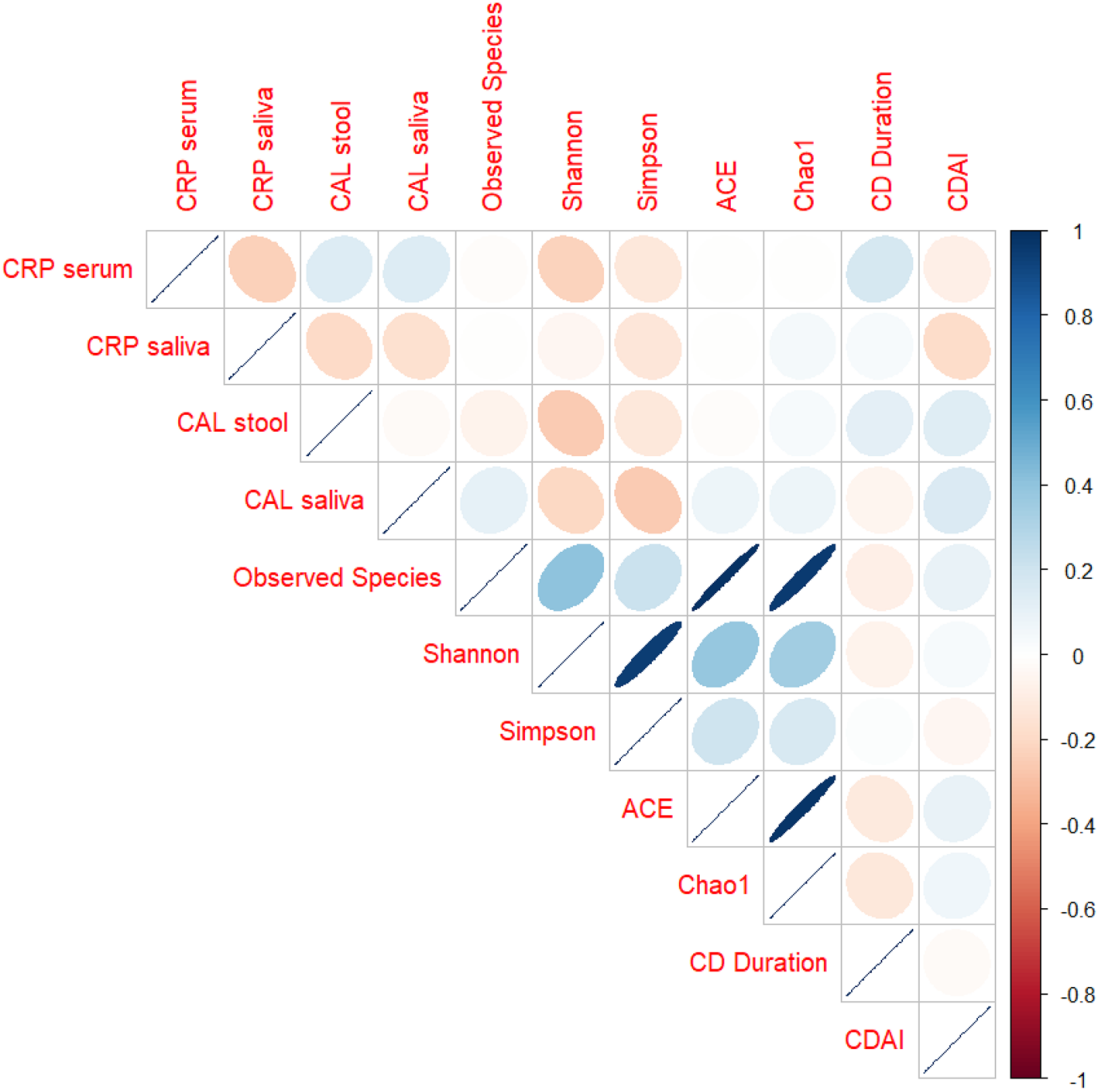
Correlation between inflammatory biomarkers, α-diversity indices, CDAI and disease duration. Blue ellipses represent positive correlation while orange ellipses represent negative correction. The intensity of color is proportional to the magnitude of the correlation. The ellipses have their eccentricity parametrically scaled to the correlation value.

Figure 7 depicts the correlation analysis of inflammatory biomarkers, α-diversity, disease duration and CDAI in CD patients. A negative relation was found between inflammatory biomarkers and α-diversity indices, in particular Shannon and Simpson indices in CD patients, reflecting an inverse relationship between salivary microbiome diversity and inflammation. Stool CAL was positively correlated with CDAI score and disease duration. Positive correlation was found between serum CRP and CD duration, and between salivary CAL with CDAI score.

## Discussion

The human oral microbiome represents a microbial-rich habitat, contributing to various oral pathologies^24^. Nowadays, it is accepted that the oral microbiome is linked to diseases outside the mouth, including gut inflammatory disorders via the oral-gut-axis^10,14^. This study confirmed that CD patients had perturbed oral microbiome and shed light on different factors probably contributing to dysbiosis. Salivary microbiome was characterized using Oxford nanopore technology by sequencing the entire 16S rRNA gene^18^, being superior to the previous research using short-read sequencing^5^. Bioinformatic analyses revealed a plethora of significant bacterial features differentiating CD from HC. Lower *Firmicutes/Bacteroidetes* (F/B) ratio was detected in CD patients. The difference from HC was non-significant, but this alteration confirms dysbiosis in CD patients. F/B ratio is widely accepted as a measure of homeostasis; thus, an increased or decreased F/B ratio is regarded as dysbiosis. Reduction in F/B ratio was reported in many IBD cases^25^. Increased abundance of *Bacteroidetes* was detected in association with elevated inflammatory cytokines and immunoglobulin A in the saliva of IBD patients^5^. A previous study confirmed that reduction in *Firmicutes* is associated with lower gut microbiota diversity in IBD patients^26^.

LDA analysis demonstrated significant differences between CD and HC. Five unique bacterial species were enriched in CD and depleted in HC, representing potential biomarkers for CD. By checking individual species, *Dolosigranulum pigrum,* from *Firmicutes*, is part of the human nasal microbiota, and acts as an immune modulator providing protection against microbial infections, and preventing colonization by pathogenic species^27^. However, it is considered an opportunistic pathogen, reported to cause eye infections, sepsis, nosocomial and ventilator-associated pneumonia^28^. *Veillonella dispar* is a Gram-negative anaerobic bacterium with lactate-fermenting ability, present in the intestine and oral mucosa. It is a nitrate-reducing bacterium in the oral cavity which is beneficially antibacterial^29^. *V. dispar* can cause severe infections such as bacteremia, infective endocarditis, prosthetic joint infections, and chronic periodontitis^30,31^. *Prevotella,* from *Bacteroidetes*, is one of the core anaerobic genera in the oral microbiome, gastrointestinal and respiratory tracts. *P. jejuni* was recognized as a novel species detected for the first time in a jejunal biopsy from a child with celiac disease, another inflammatory bowel condition^32^. It is among the resident oral flora, with other species of *Prevotella*^33^*.* There are studies suggesting that a higher level of *Prevotella* can be a potential indicator of CD^34^. *Prevotella* species often occur in opportunistic infections and dysbiosis-associated disease, and produce major metabolic end-products such as acetic and succinic acids^32^. Furthermore, perturbation of the gut microbiome by some *Prevotella spp.* was shown to enhance host susceptibility to mucosal inflammation in animal models via reduction of IL-18 production^35^. *Megasphaera stantonii,* from *Firmicutes,* is an obligately anaerobic Gram-negative, butyrate-producing bacterium^36^. In general, butyrate-producing bacteria have health-promoting activity by maintaining epithelial barrier function and integrity, and inhibiting inflammation^37^. As for *Lactobacillus backii*, no report was found in human, but this organism was reported to have a beer-spoiling effect^38^. Noteworthy, many species were found more abundant in HC but depleted in CD, reflecting the significant difference between the groups, and confirming dysbiosis in CD patients.

CD patients demonstrated poorer oral health than HC, in agreement with previous reports of a higher prevalence of dental caries and periodontitis in CD patients^39,40^. However, the previous studies did not analyze the oral microbiota in CD patients and lacked any evidence of dysbiosis in these patients. In CD, many significant features were detected, especially at the species level in patients with different oral health status. Highly cariogenic and periopathogenic species were detected, as *Fusobacterium periodonticum, Lactobacillus fermentum*, *Lactobacillus acidophilus* and *Streptococcus mutans*, in agreement with previous studies reporting poor dental health with more pathobionts in the salivary microbiota, including *S. mutans* and* P. gingivalis*^41^*.* In CD patients with good oral health, multiple significant bacteria were detected as *Porphyromonus gingivalis, Prevotella jejuni, Prevotella dentalis, Tanerella forsythia,* and *Bacteroides fragilis.* It is noteworthy that many of these bacteria are pathogens or opportunistic pathogens^42^, indicating that oral dysbiosis can occur in CD patients even in the absence of oral pathology.

Besides oral health, we also explored different potential factors that might have contributed to dysbiosis, including factors related to CD, specifically medications, disease activity, duration, and frequency of relapse of symptoms. Among these factors, medications had the most significant effect on the oral microbiome. A sole bacterium was found significantly more in patients receiving only infliximab which is *Simonsiella muelleri,* from *Betaproteobacteria* belonging to *Neisseriaceae*^43^*.* No previous studies were found on this species in relation to any disease. On the other hand, concurrent use of monoclonal antibodies, steroids and immunosuppressants was associated with the emergence of multiple pathogenic and opportunistic bacteria from *Proteobacteria* such as *Escherichia coli, Salmonella enterica, Klebsiella pneumoniae, Pseudomonas aeruginosa,* and *Enterobacter cloacae,* that are associated with serious infections. Previous studies confirmed the link between *Proteobacteria* and IBD as these bacteria have adherent and invasive properties^44^, exploiting host defenses, and leading to proinflammatory changes, with gut microbiota alteration causing dysbiosis^45^. *E. coli*, in particular adherent invasive strains, had been linked to IBD pathogenesis by abnormal colonization of gut mucosa via the interaction of the mannose-specific adhesin FimH of type 1 pili with mannosylated proteins on the epithelial cell surface^46,47^. Thus, some studies recommended the use of anti-adhesive compounds against these invasive *E. coli,* with promising results in vitro and in vivo^48^. Such initiatives open the door for the development of microbiome-targeted interventions, which can influence the natural course of IBD. The exact mechanisms that lead to the increase in *Proteobacteria* in IBD are still unknown, but the most accepted explanation is the oxygen hypothesis, suggesting the depletion of obligate anaerobes and enrichment of facultative anaerobes, from *Proteobacteria* such as *Enterobacteriaceae,* during inflammation^49^. A previous study confirmed that multidrug-resistant *Klebsiella* tend to colonize when the intestinal microbiota is dysbiotic causing a serious gut inflammatory response in genetically vulnerable hosts^50^. Pathobionts stored in the oral cavity can aggravate intestinal disease and affect healthy gut microbiota which offer colonization resistance against pathogenic bacteria^50^. Another study reported high levels of antibodies against *Klebsiella* in CD patients documented by multiple gastroenterology clinics in the UK^51^. In numerous situations, CD patients' antibody response to *Klebsiella* bacteria was significantly higher than those of healthy individuals. As a result, it is conceivable that CD could be brought on by persistent, subclinical infections of the large bowel with *Klebsiella*, which would then cause inflammation and tissue damage due to the binding of anti-*Klebsiella* and anti-self-tissue antibodies to the cross-reactive targeted antigens^51^. Therefore, this interplay proves that the immune response to pathogenic bacteria either in the gut or oral cavity can be implicated in IBD pathogenesis.

When the activity of CD was considered, three-quarters of the patients had inactive disease, with an increased abundance of multiple species of *Capnocytophaga*, that have been reported to cause granulomatous inflammation mimicking CD^52^. Patients with inactive disease also had an increased abundance of *Fusobacterium ulcerans*, which was associated with lymphovascular invasion in patients with colorectal cancer^53^, but was not reported before in IBD patients. Patients with active disease had a significant increase in *Lactobacilli,* which are part of the normal microbiota. Both lactate-producing and lactate-utilizing bacteria coexist in the human intestine, contributing to gut health^54^.

As for disease duration, multiple species were significantly different mainly in newly diagnosed CD patients, including oral periopathogens as *Prophyromonas gingivalis* and cariogenic bacteria as *Streptococcus viridans.* This is interesting as this may reflect that these oral pathogens are important contributors to dysbiosis, at early stages of the disease. A previous study indicated that colonization of several oral pathogens, including a subset of *Porphyromonas gingivalis*, *Streptococcus mutans*, *Fusobacterium nucleatum*, among others may result in intestinal epithelial barrier destruction, excessive secretion of inflammatory cytokines, disruption of the host immune system, and gut dysbiosis^55^. Noteworthy, some pathogenic bacteria such as *Klebsiella pneumoniae* were enriched in patients having the disease for more than a decade. As stated, this bacterium has been linked to inflammatory changes in CD^50^. The duration of the disease gives us an insight into microbiota shifts throughout the years.

Patients were also differentiated based on the frequency of relapses. Patients with more frequent relapses had an increased abundance of *Prevotella* as *P. oris* and* P. jejuni.* Another notable bacterial species was *Simonsiella muelleri*, which was also significantly increased by the use of monoclonal antibodies in our study. Patients with less frequent relapses harbored multiple health-promoting bacteria from *Clostridium, Lactobacillus* and *Ruminococcus.* Multiple species of *Clostridium* and *Lactobacillus*, are commensal organisms predominant in the healthy gut, with lots of salutary effects on homeostasis with a huge potential as probiotics^56^. *Ruminococcus* serves to degrade and convert complex polysaccharides into a variety of nutrients for host use^57^. *Clostridia* and *Ruminaceae*, are known to produce short-chain fatty acids, particularly butyrate, required for orchestrating multiple physiological functions to maintain gut health^57^. It is expected that members of the *Lactobacillales* and *Clostridiales* play a significant role in upcoming efforts to restore the functionality of the microbiota after disruption because significant positive functions have been attributed to these groups^58^. Thus, the existence of these bacteria in CD patients with controlled disease suggests their contribution to microbiota stability in the absence of inflammation.

Significantly altered species were compared using a Venn diagram to study the link between various factors. It was obvious that the effect of each factor is unique, as very few species were shared indicating that each factor has its distinct impact on the microbiome, which was altered by the combined effect of all the factors collectively contributing to dysbiosis. This was further confirmed when a heatmap was constructed revealing unique microbiota signatures in CD patients grouped based on the five factors. Nevertheless, the most striking variations in terms of microbiota enrichment and depletion were obvious in patients segregated based on oral health status and medications, confirming their significant impact on the oral microbiome in CD patients. It is worth noting that most CD patients had poor oral health. This clarifies the variation in oral microbiota signatures among CD patients, that is probably attributed to the local effect of oral infection and inflammation on the environment in the oral cavity of CD patients, favoring overgrowth of more pathogenic and opportunistic organisms as reported in our study. Most of the previous studies compared the microbiota in CD and HC, without considering patients’ oral health and disease related factors; thus, failed to explain the differences among groups. On the other hand, our analysis of shared microbiota and microbiome signature influenced by various factors revealed interesting facts that could explain some of the variations. For instance, some species that were higher in CD than HC were related to important disease parameters as *Prevotella jejuni* associated with high relapse rate, *Lactobacillus backii* associated with less relapses, and *Dolosigranulum pigrum* associated with periodontal disease. These findings added power to our study, as we were able to explain the variations between CD and HC based on the contribution of each factor.

Significantly lower α-diversity was seen in CD compared to HC with depletion of beneficial bacteria indicating dysbiosis^59^. CD patients with both caries and periodontitis had the lowest α-diversity, reflecting the effect of oral health on oral microbiota. β-diversity was not significantly different between CD and HC, and even among CD patients grouped according to different factors. A controversial previous study revealed significantly higher β-diversity heterogeneity in the IBD group^14^.

As for inflammatory biomarkers, C-reactive protein (CRP) and calprotectin (CAL) were analyzed. CRP is a protein released by the liver in inflammatory conditions such as CD. Calprotectin is a calcium-binding neutrophil protein that is stable during intestinal transit. CAL is strongly correlated with endoscopic and histological CD activity and its high fecal levels reflect intestinal inflammation^60^. Interestingly, correlation analysis revealed an inverse relationship between salivary microbiome diversity and inflammatory biomarkers. CAL seems to be superior to CRP as fecal CAL was positively correlated with CDAI score and disease duration, while salivary CAL correlated with CDAI score, suggesting a strong relation to disease activity, as reported before^60^. Our findings suggest that higher degrees of gut inflammation are associated with less α-diversity indicating dysbiosis and depletion of beneficial microbiota from the oral cavity of CD patients. Thus, oral health might have impacted gut health. This can be explained by the fact that oral bacteria can surely translocate into the intestine as each person can swallow 1–1.5 L of saliva per day^61^. Swallowed pathogenic oral microbes have the potential to disrupt the balance of gut microbiota. Microbial metabolites might be involved as they can be swallowed or absorbed, which can be another hypothesis explaining how oral dysbiosis can affect the gut^61^. The presence of pathogenic bacteria in the oral cavity of CD is alarming, since it can enter the bloodstream contributing to systemic inflammation^62^. Ectopic colonization of oral bacteria may lead to destruction of the intestinal epithelial barrier, secretion of inflammatory cytokines, disruption of the host immune system, and dysbiosis of gut microbiota, consequently aggravating chronic intestinal inflammation^63^. Inflammation may begin in the oral cavity and spread to the gut as pathobionts move between those body sites^64^. Translocation of oral microbiota to the gut may be a common feature of microbial dysbiosis which is a signature of CD^65^. A recent study in mice has shown that inflammation of the oral mucosa combined with overgrowth of pathobionts in the oral microbiota led to colitis via gut colonization and the induction and migration of bacteria-reactive T cells (Th17) to the gut. Thus, oral inflammation can cause exacerbation of inflammation by supplying the gut with both pathobionts and pathogenic T cells^66^. Another study reported that inoculation of saliva from children with CD to germ-free mice led to the accumulation of inflammatory IFN-γ^+^ CD4^+^ TH1 cells in the intestinal lamina propria and enrichment of *Fusobacterium, Veillonella* and *Klebsiella spp.* in the fecal microbiome^50^. It is noteworthy that oral pathobionts do not colonize the gut of healthy individuals; thus, patients with IBD are at higher risk of intestinal dysbiosis as a consequence of oral dysbiosis. These findings strengthen the links between the oral–gut axis, oral microbiome and immune-mediated mechanisms in IBD development.

This study had some limitations related to the small sample size which was attributed to the difficulty in recruiting both CD patients and healthy controls due to the restrictions during the COVID-19 pandemic. However, the sample size in this study is comparable to similar studies exploring the oral microbiome of IBD patients^67^. Despite these limitations, the use of long-read sequencing technology for microbiome profiling increased the value of this study by revealing the microbiota alterations down to the species level and identifying peculiar microbiota linked to multiple disease factors. Overall, this study is an important stride for more comprehensive validation studies in the future with larger cohorts of patients.

## Conclusions

Salivary dysbiosis was deciphered in a group of CD patients, with the recognition of key species enriched in CD and depleted in HC. Oral health status produced unique signatures in CD patients and seems to be an important factor aggravating dysbiosis. Multiple factors including medications were associated with the emergence of pathogenic and opportunistic species in CD patients. This is alarming, as they can be translocated into the intestine aggravating inflammation. Thus, more attention must be given to the oral health of CD patients to prevent and treat dysbiosis. Microbiota modulation might be attempted in the future to restore the balance in the oral cavity, by increasing the beneficial microbiota and getting rid of the pathogenic ones with the potential to induce inflammation. This can be a promising therapeutic approach for CD management, especially after the identification of the species that are enriched or depleted in order to use them as future therapeutic targets. Finally, this study was the first of its kind in the United Arab Emirates, expanding our knowledge of IBD, CD and the oral microbiome in the Arab world which is generally understudied.

### Supplementary Information

Supplementary Figure S1.

Supplementary Information 2.

Supplementary Information 3.

## Data Availability

Data was deposited in the Sequence Read Archive (NCBI) (SUB12496940) under BioProject (PRJNA948338): https://www.ncbi.nlm.nih.gov/bioproject/948338. This Sequence Read Archive (SRA) submission will be released on 2024-12-31 or upon publication, whichever is first. This is the link for full access to the data provided by NCBI for the reviewers: https://dataview.ncbi.nlm.nih.gov/object/PRJNA948338?reviewer=k5elp6sah726o70m63a39ndsdu.
