# Supplementary material for: Deciphering salivary microbiome signature in Crohn’s disease patients with different factors contributing to dysbiosis
Source: Sci Rep. 2023 Nov 6;13:19198. doi: 10.1038/s41598-023-46714-8 (PMC10628307; doi:10.1038/s41598-023-46714-8)
Supplement: Supplementary file 1 — Supplementary Figure S1. [file 41598_2023_46714_MOESM1_ESM.docx]

**Figure S1.** Significant features at the genus level detected using LDA in CD patients based on variation in oral health status (A), IBD drugs (B), disease duration (C), activity (D), and frequency of relapses. Genera with LDA score>2 are shown

**A**

**B**

**C**

**D**

**

**E**
