# Supplementary material for: Deciphering salivary microbiome signature in Crohn’s disease patients with different factors contributing to dysbiosis
Source: Sci Rep. 2023 Nov 6;13:19198. doi: 10.1038/s41598-023-46714-8 (PMC10628307; doi:10.1038/s41598-023-46714-8)
Supplement: Supplementary file 2 — Supplementary Information 2. [file 41598_2023_46714_MOESM2_ESM.docx]

Alpha diversity in CD patients with different factors that might contribute to dysbiosis.

1. **IBD medications**

When different indices of alpha diversity were compared in CD subjects based on IBD drug use. The p values were not significant, Chao1, Simpson, Shannon, observed and ACE indices. (p value: 0.91829, 0.81776, 0.53979, 0.6 and 0.638 respectively).

**A. B. C.**

**D. E.**

**Figure S2. Alpha diversity indicies** **for IBD medications: A. Chao1, B. Shannon, C. Simpson, D. observed, and E.ACE**

1. **Activity of disease**

When different indices of alpha diversity were compared in CD subjects based on the activity of the disease. The p values were not significant, Chao1, Shannon, Simpson, observed and ACE indices (p value: 0. 823, 0.44036, 0.265, 0.97 and 0.849 respectively).

**A. B. C.**

**D. E.**

**Figure S3. Alpha diversity indicies for activity of disease A. Chao1, B. Shannon, C. Simpson, D. observed, and E. ACE.**

1. **Frequency of relapse of symptoms**

When different indices of alpha diversity were compared with CD subjects based on the relapse of symptoms. The p values were not significant, Chao1, Shannon, Simpson, observed and ACE indices (p value: 0.534, 0.898, 0.9926, 0.915 and 0.88 respectively).

**A. B. C.**

******

**D. E.**

******

**Figure S4. Alpha diversity indicies for frequency of relapse of symptoms A. Chao1, B. Shannon, C. Simpson, D. observed, and E. ACE.**

1. **Duration of disease**

When different indices of alpha diversity were compared CD subjects based on the duration of the disease. The p values were not significant, Chao1, Shannon, Simpson, observed and ACE indices. (p value: 0.763, 0.754,0.428, 0.53and 0.659 respectively).

**A. B. C.**

**D. E.**

**Figure S5. Alpha diversity indicies for duration of disease A. Chao1, B. Shannon, C. Simpson, D. observed, and E. ACE.**
