# Supplementary material for: Deciphering salivary microbiome signature in Crohn’s disease patients with different factors contributing to dysbiosis
Source: Sci Rep. 2023 Nov 6;13:19198. doi: 10.1038/s41598-023-46714-8 (PMC10628307; doi:10.1038/s41598-023-46714-8)
Supplement: Supplementary file 3 — Supplementary Information 3. [file 41598_2023_46714_MOESM3_ESM.docx]

Beta diversity in CD patients with different factors that might contribute to dysbiosis.

Bray Curtis and Jaccard were used as the distance methods. Clustering was demonstrated using principles coordinates analysis (PcoA) as a data comparison technique to visualize sample similarity based on different factors such as:

**1 -Oral health (figure S6-A)**

P value (0.276) indicates non-significant difference between the groups. PCoA shows clustering of samples mostly within periodontal disease, then periodontal disease and caries, with a very wide circle of caries, indicating high variability.

**2 -IBD drugs (figure S6-B)**

P value (0.379) indicates non-significant difference between the groups. All samples are grouped in the biologicals circle since most of patients were consuming this type of medication, but 2 samples were out of the red circle and into the blue circle of biologicals and steroids.

**3 -Activity of the disease (figure S6-C)**

P value (0. 886) indicates non-significant difference between the groups. All samples except one overlapped, the outcast one is a patient with active disease.

**4 -Frequency of relapse of symptoms (figure S6-D)**

P value (0.4) indicates non-significant difference between the groups. Samples are gathered with some samples of patients within 0-1 relapses per year outside and only one away for a patient with a relapse of more than 2 times per year.

**5 -Duration of the disease (figure S6-E)**

P value (0.753) indicates non-significant difference between the groups. Only two samples cluster apart which are patients with duration of the disease lasting 1-10 years, while the rest overlapped with disease duration of more than 10 years.

**A**


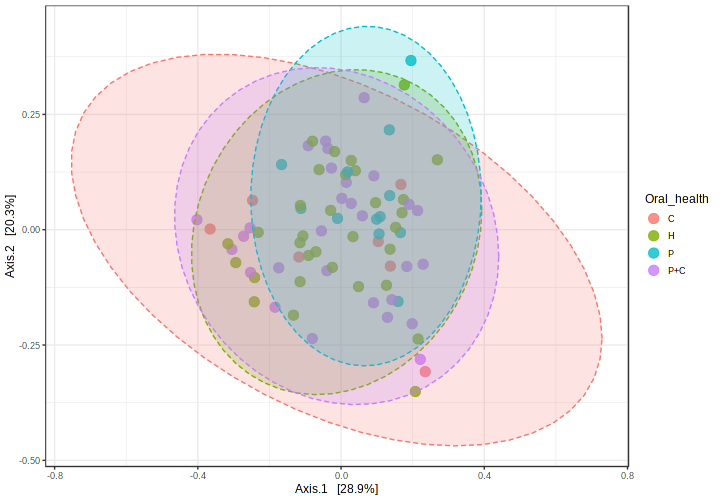


**B**

**
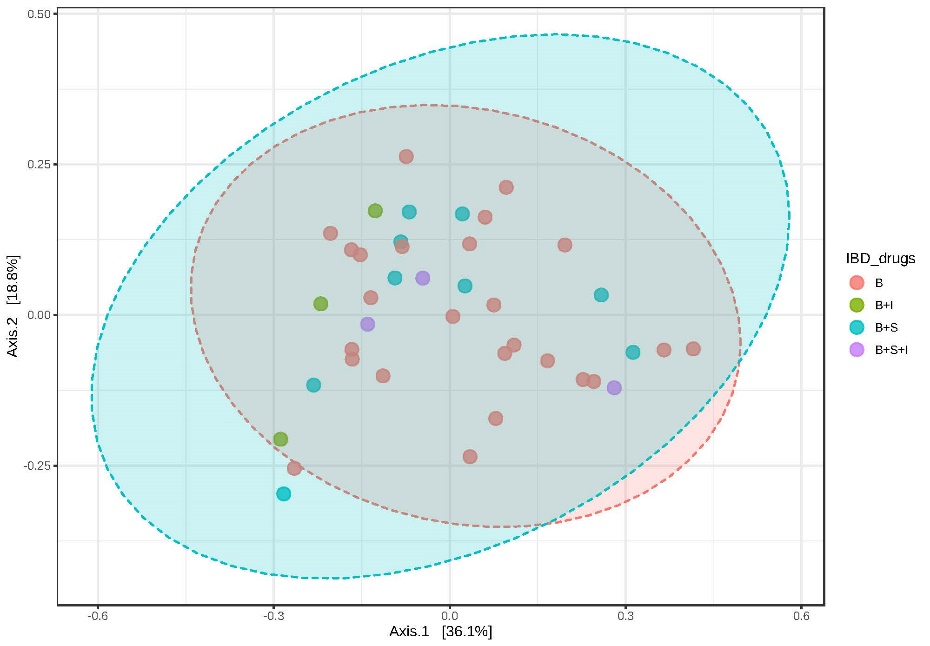
**

**C**

**
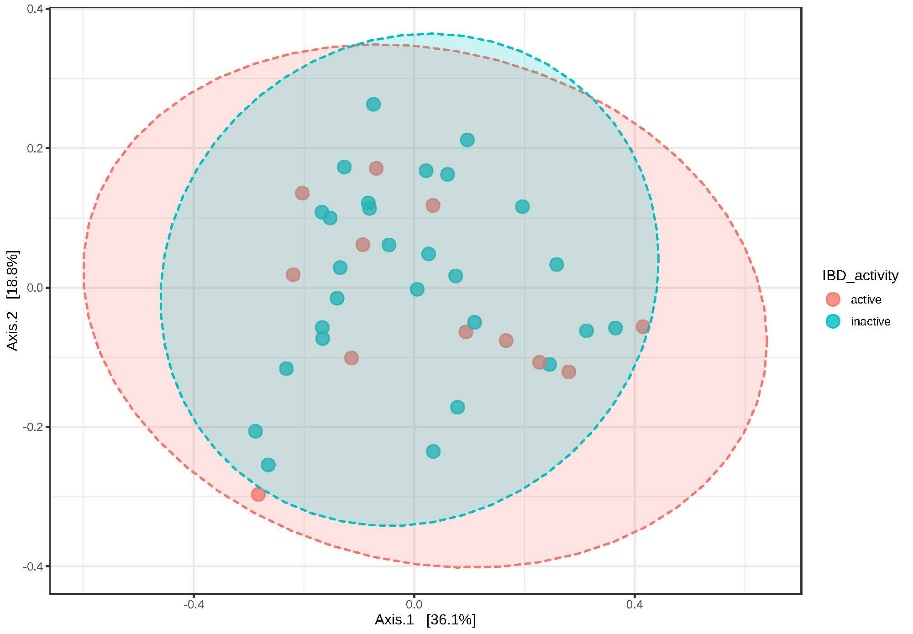
**

**D**


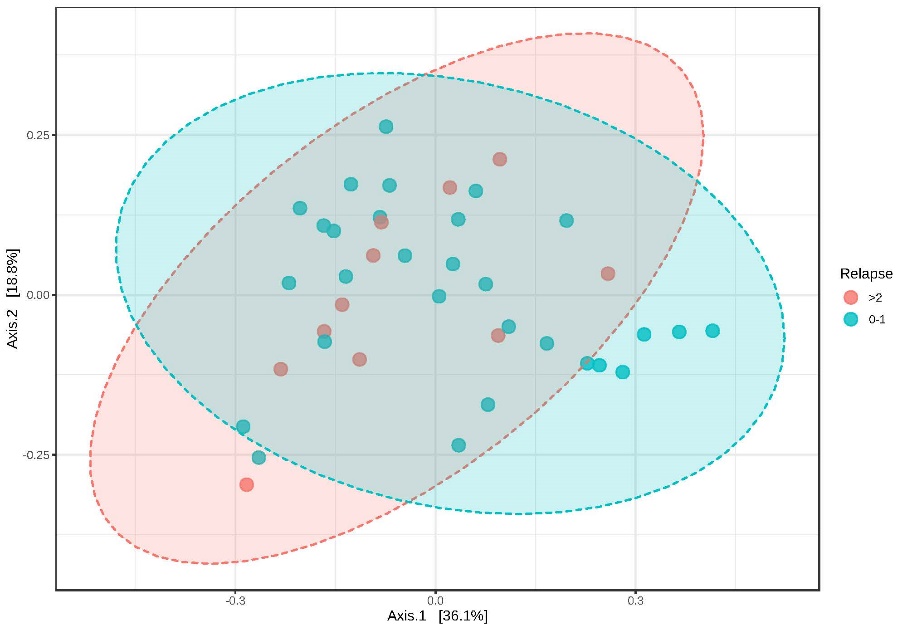


**E**


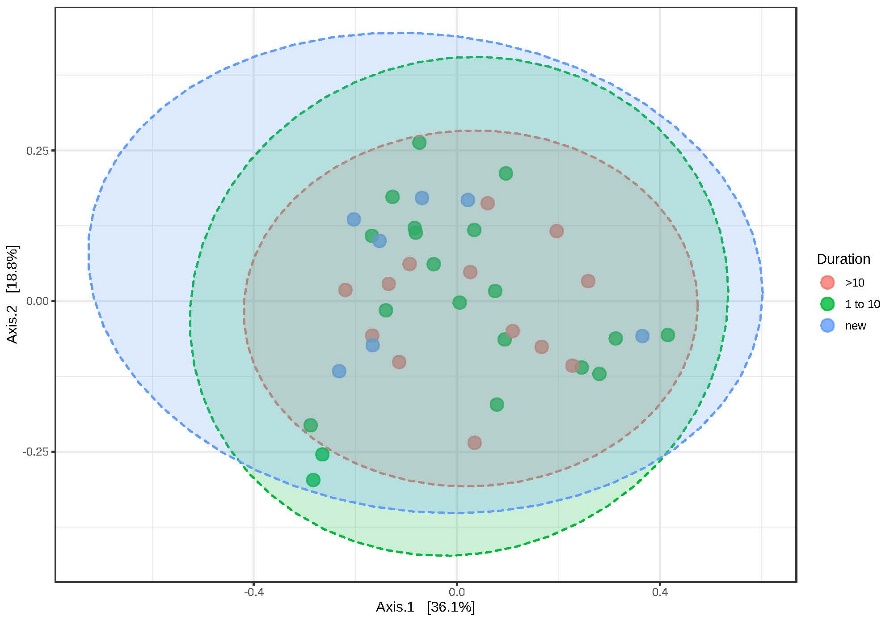


**Figure S6.** **Beta diversity using PERMANOVA as the statistical method. A-oral health; B- IBD drugs; C- activity of the disease; D- frequency of relapse of symptoms and E-duration of the disease.**
